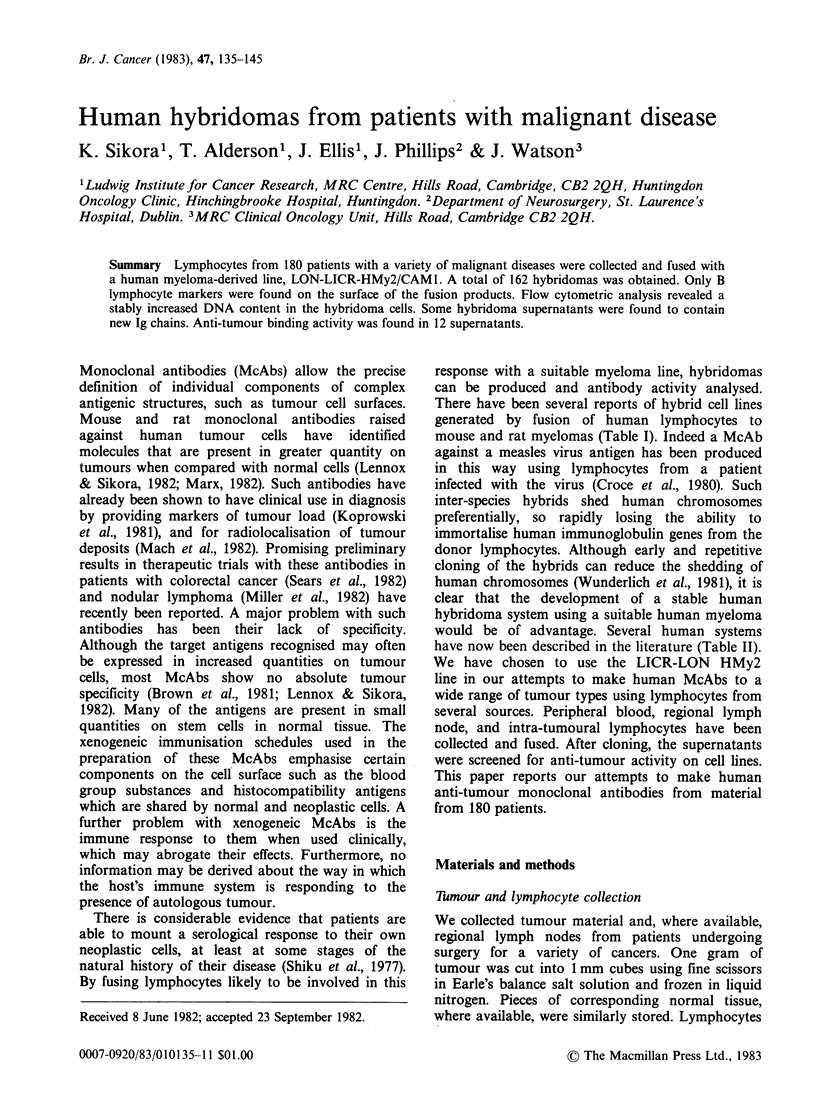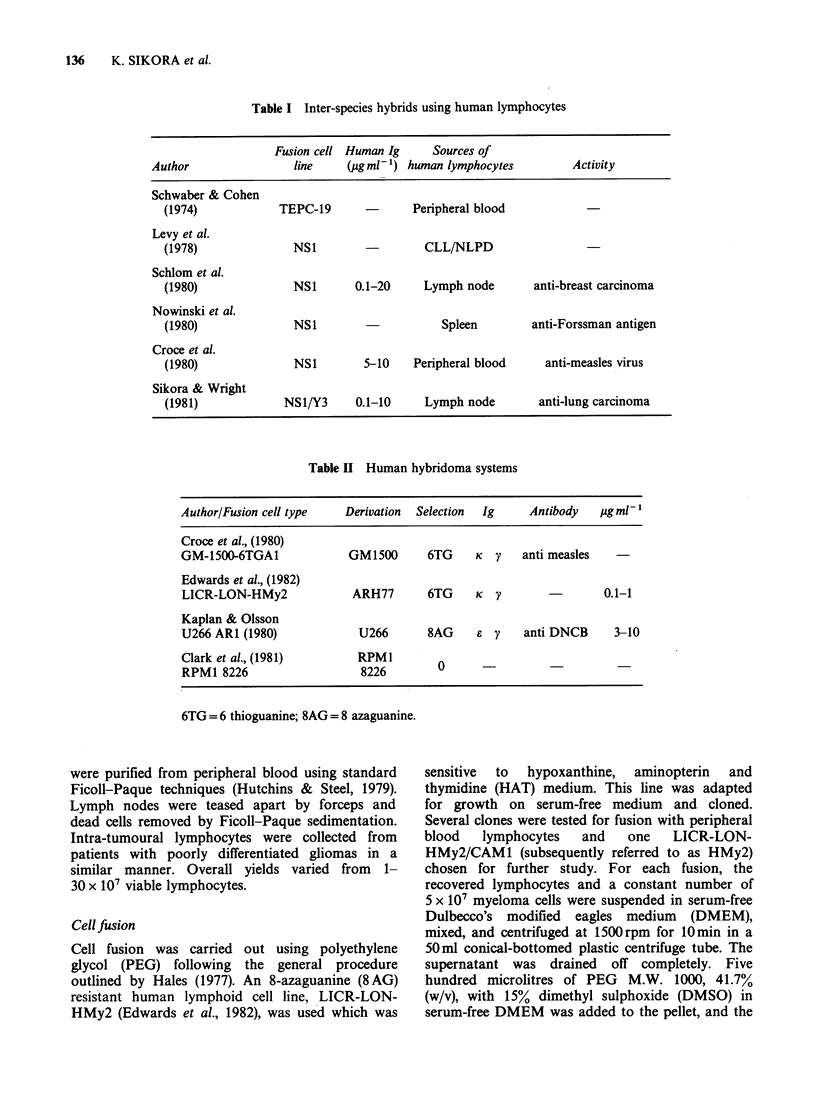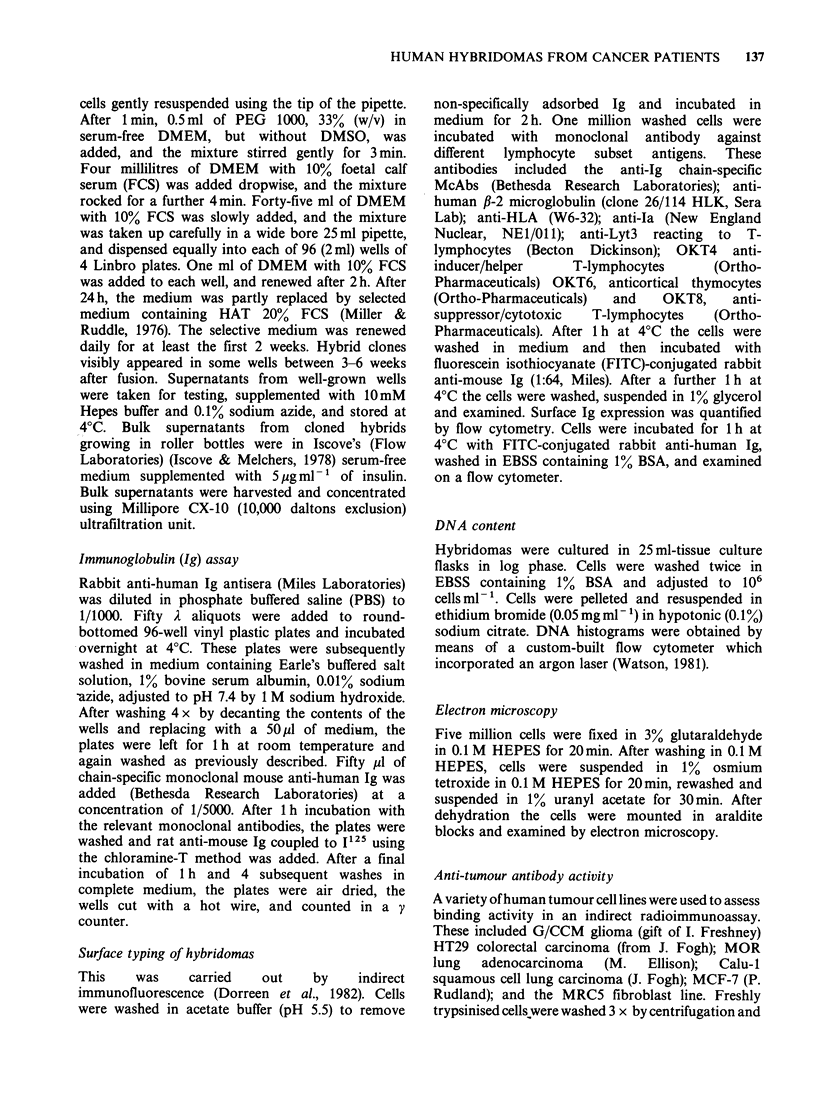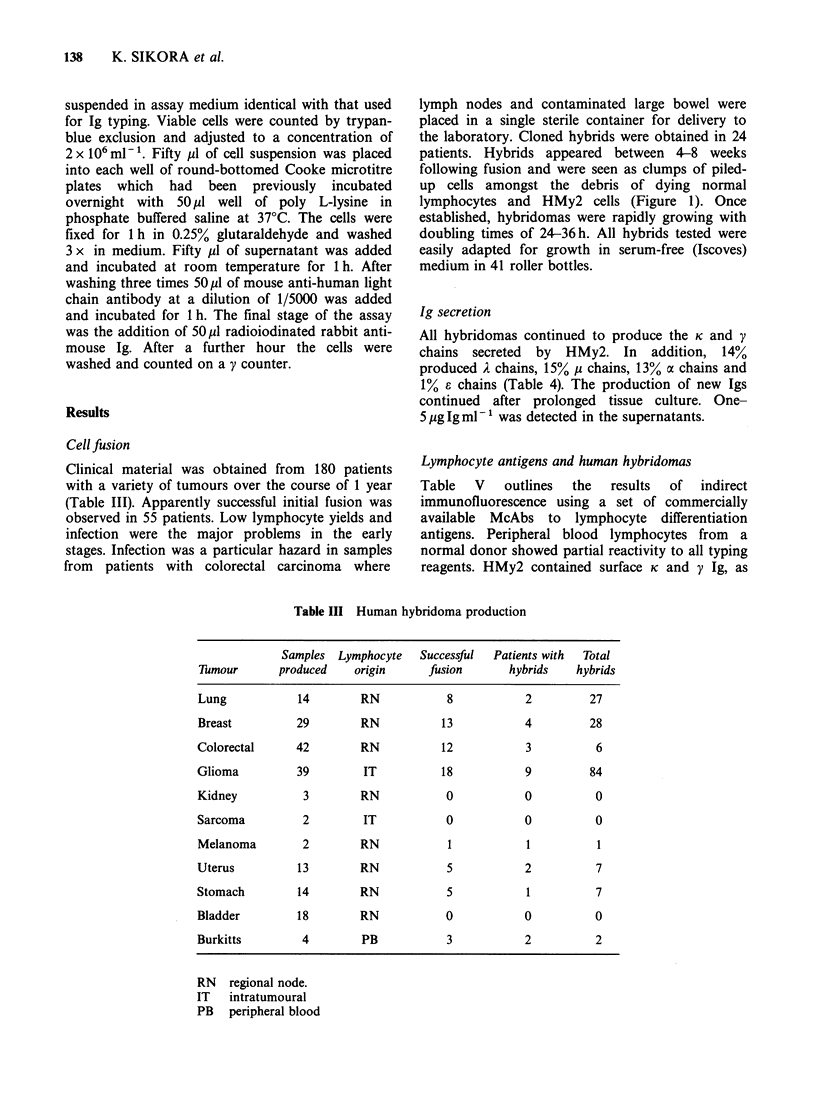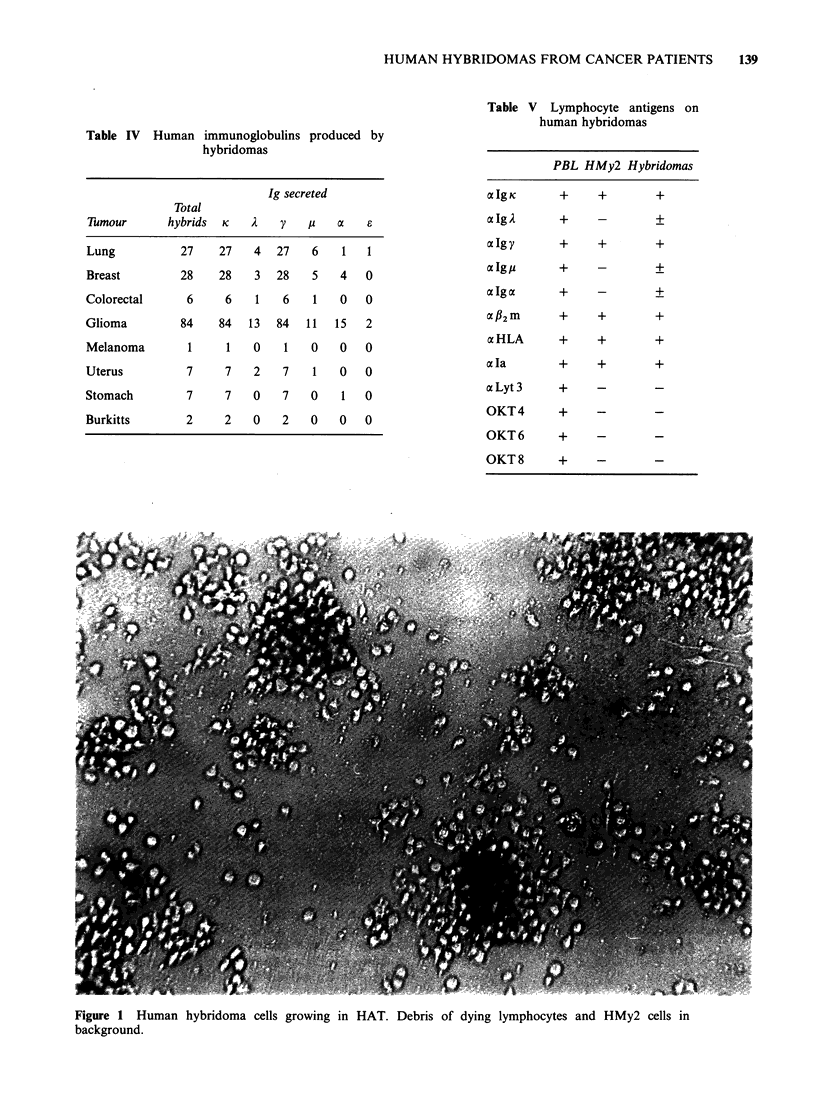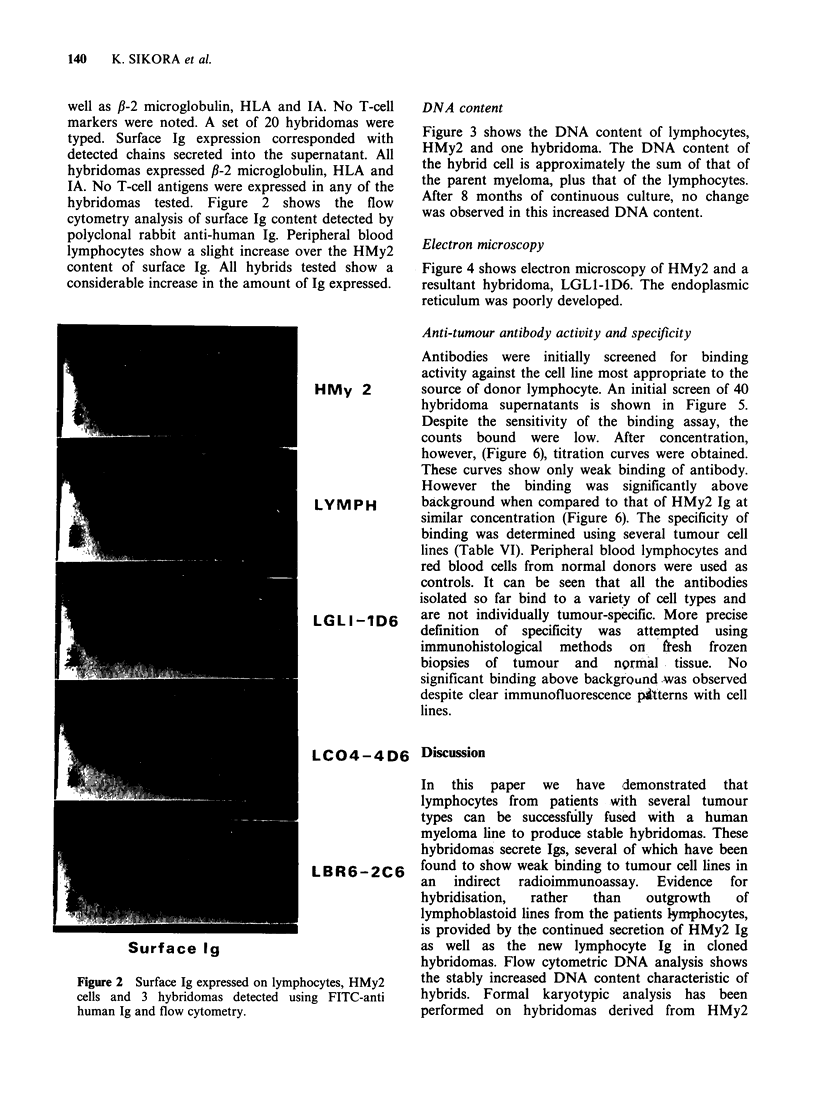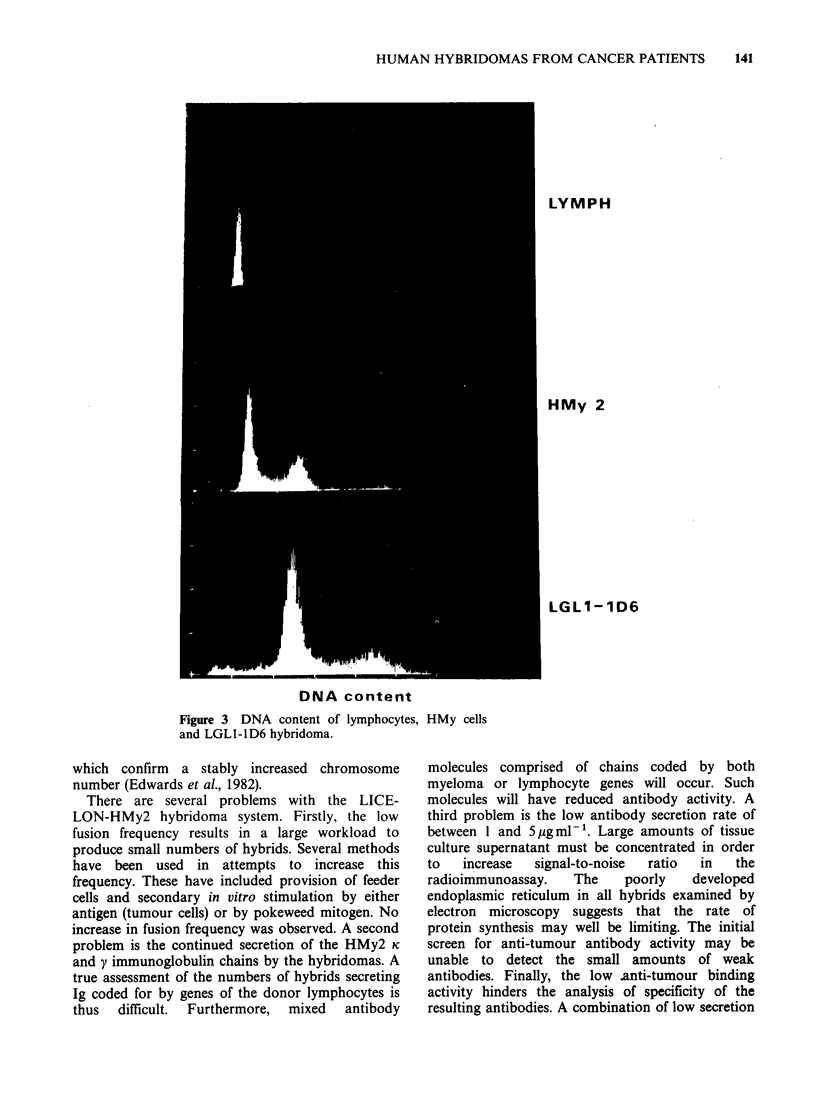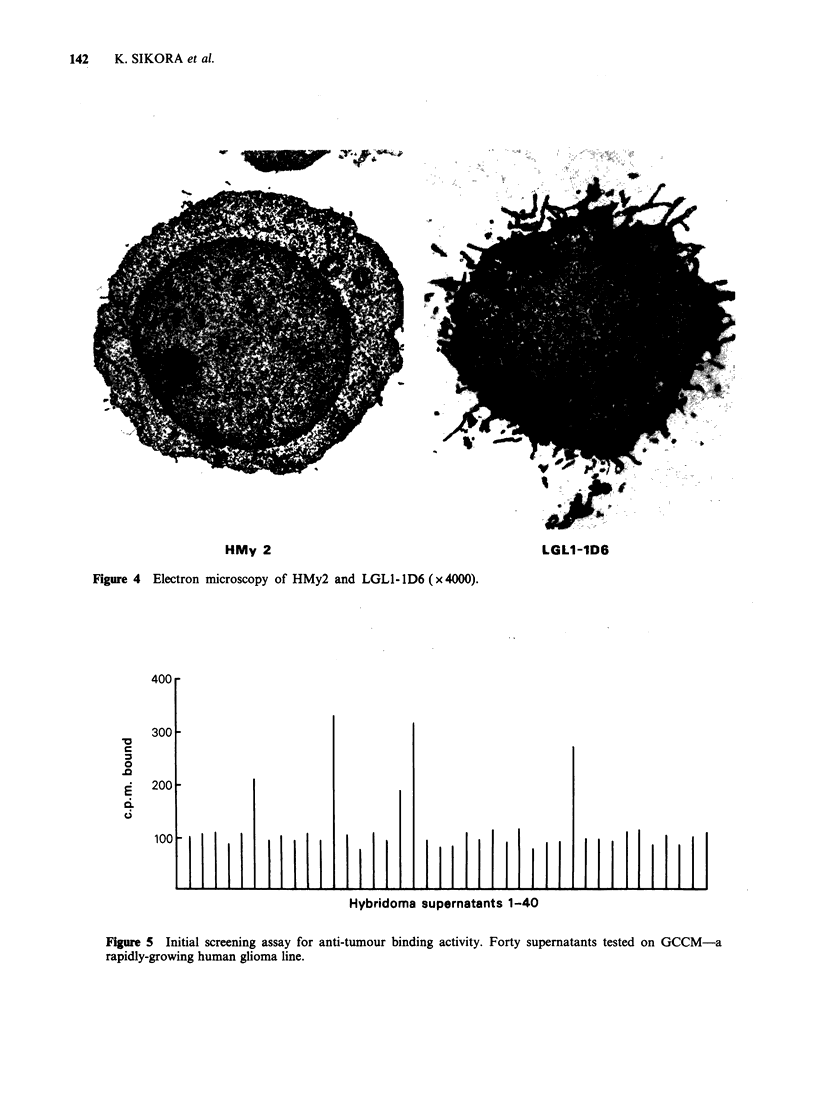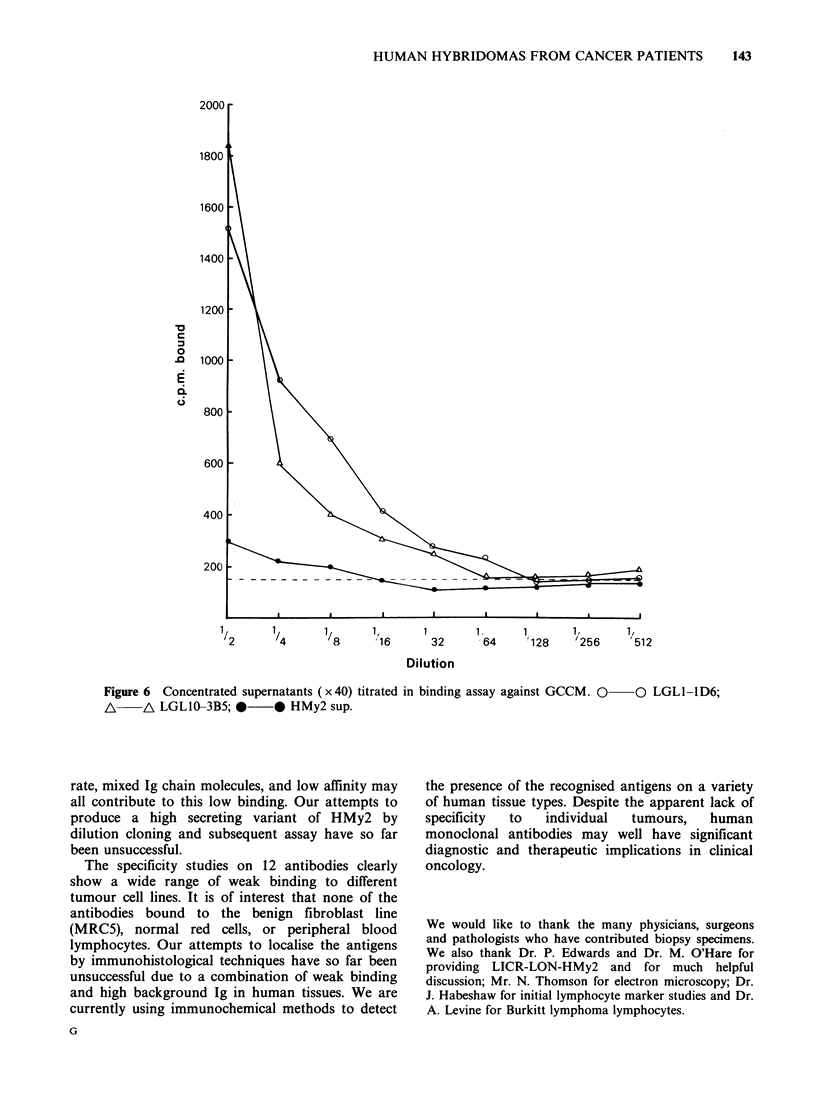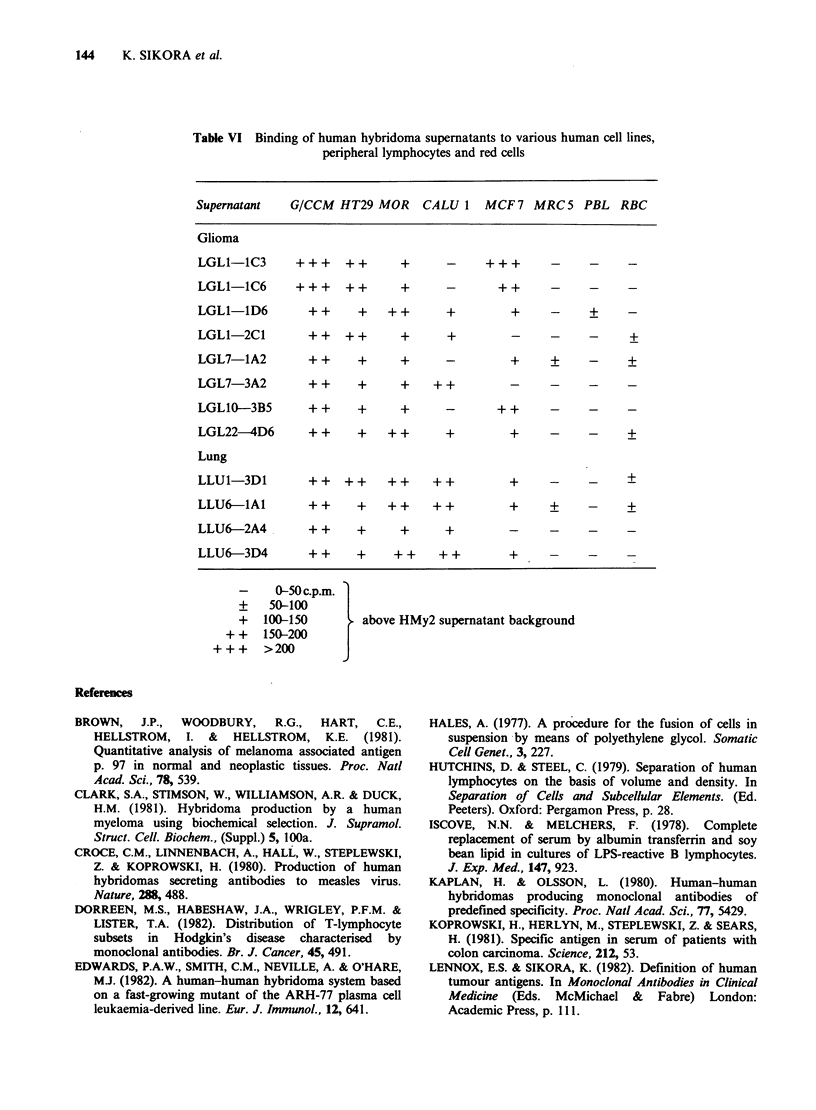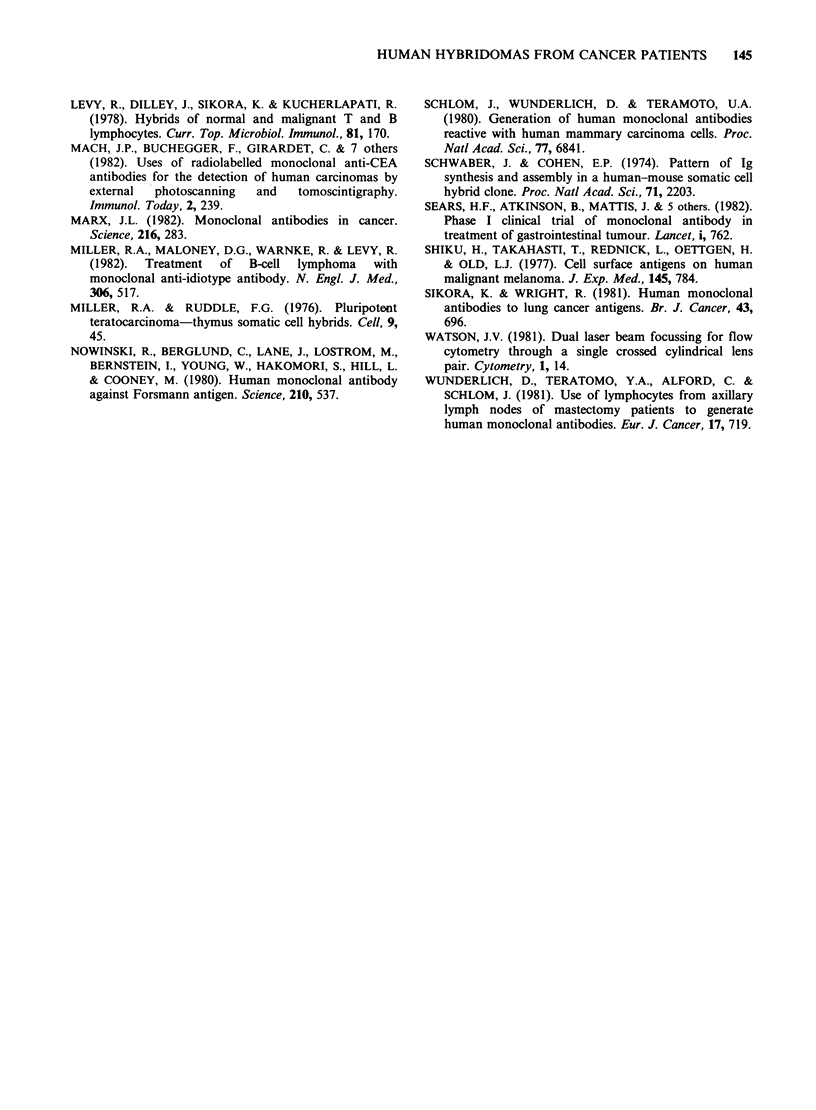# Human hybridomas from patients with malignant disease.

**DOI:** 10.1038/bjc.1983.16

**Published:** 1983-01

**Authors:** K. Sikora, T. Alderson, J. Ellis, J. Phillips, J. Watson

## Abstract

**Images:**


*Br. J. Cancer* (1983), **47**, 135–145

### Human hybridomas from patients with malignant disease

K. Sikora[1], T. Alderson[1], J. Ellis[1], J. Phillips[2] & J. Watson[3]

[1]*Ludwig Institute for Cancer Research, MRC Centre, Hills Road, Cambridge, CB2 2QH, Huntingdon Oncology Clinic, Hinchingbrooke Hospital, Huntingdon.* [2]*Department of Neurosurgery, St. Laurence's Hospital, Dublin.* [3]*MRC Clinical Oncology Unit, Hills Road, Cambridge CB2 2QH.*

**Summary** Lymphocytes from 180 patients with a variety of malignant diseases were collected and fused with a human myeloma-derived line, LON-LICR-HMy2/CAM1. A total of 162 hybridomas was obtained. Only B lymphocyte markers were found on the surface of the fusion products. Flow cytometric analysis revealed a stably increased DNA content in the hybridoma cells. Some hybridoma supernatants were found to contain new Ig chains. Anti-tumour binding activity was found in 12 supernatants.

Monoclonal antibodies (McAbs) allow the precise definition of individual components of complex antigenic structures, such as tumour cell surfaces. Mouse and rat monoclonal antibodies raised against human tumour cells have identified molecules that are present in greater quantity on tumours when compared with normal cells (Lennox & Sikora, 1982; Marx, 1982). Such antibodies have already been shown to have clinical use in diagnosis by providing markers of tumour load (Koprowski *et al.*, 1981), and for radiolocalisation of tumour deposits (Mach *et al.*, 1982). Promising preliminary results in therapeutic trials with these antibodies in patients with colorectal cancer (Sears *et al.*, 1982) and nodular lymphoma (Miller *et al.*, 1982) have recently been reported. A major problem with such antibodies has been their lack of specificity. Although the target antigens recognised may often be expressed in increased quantities on tumour cells, most McAbs show no absolute tumour specificity (Brown *et al.*, 1981; Lennox & Sikora, 1982). Many of the antigens are present in small quantities on stem cells in normal tissue. The xenogeneic immunisation schedules used in the preparation of these McAbs emphasise certain components on the cell surface such as the blood group substances and histocompatibility antigens which are shared by normal and neoplastic cells. A further problem with xenogeneic McAbs is the immune response to them when used clinically, which may abrogate their effects. Furthermore, no information may be derived about the way in which the host's immune system is responding to the presence of autologous tumour.

There is considerable evidence that patients are able to mount a serological response to their own neoplastic cells, at least at some stages of the natural history of their disease (Shiku *et al.*, 1977). By fusing lymphocytes likely to be involved in this response with a suitable myeloma line, hybridomas can be produced and antibody activity analysed. There have been several reports of hybrid cell lines generated by fusion of human lymphocytes to mouse and rat myelomas (Table I). Indeed a McAb against a measles virus antigen has been produced in this way using lymphocytes from a patient infected with the virus (Croce *et al.*, 1980). Such inter-species hybrids shed human chromosomes preferentially, so rapidly losing the ability to immortalise human immunoglobulin genes from the donor lymphocytes. Although early and repetitive cloning of the hybrids can reduce the shedding of human chromosomes (Wunderlich *et al.*, 1981), it is clear that the development of a stable human hybridoma system using a suitable human myeloma would be of advantage. Several human systems have now been described in the literature (Table II). We have chosen to use the LICR-LON HMy2 line in our attempts to make human McAbs to a wide range of tumour types using lymphocytes from several sources. Peripheral blood, regional lymph node, and intra-tumoural lymphocytes have been collected and fused. After cloning, the supernatants were screened for anti-tumour activity on cell lines. This paper reports our attempts to make human anti-tumour monoclonal antibodies from material from 180 patients.

### Materials and methods

### *Tumour and lymphocyte collection*

We collected tumour material and, where available, regional lymph nodes from patients undergoing surgery for a variety of cancers. One gram of tumour was cut into 1 mm cubes using fine scissors in Earle's balance salt solution and frozen in liquid nitrogen. Pieces of corresponding normal tissue, where available, were similarly stored. Lymphocytes

Received 8 June 1982; accepted 23 September 1982.

0007-0920/83/010135–11 $01.00

© The Macmillan Press Ltd., 1983

**Table I** Inter-species hybrids using human lymphocytes

| *Author* | *Fusion cell line* | *Human Ig* ($\mu g\ ml^{-1}$) | *Sources of human lymphocytes* | *Activity* |
|---|---|---|---|---|
| Schwaber & Cohen (1974) | TEPC-19 | — | Peripheral blood | — |
| Levy *et al.* (1978) | NS1 | — | CLL/NLPD | — |
| Schlom *et al.* (1980) | NS1 | 0.1–20 | Lymph node | anti-breast carcinoma |
| Nowinski *et al.* (1980) | NS1 | — | Spleen | anti-Forssman antigen |
| Croce *et al.* (1980) | NS1 | 5–10 | Peripheral blood | anti-measles virus |
| Sikora & Wright (1981) | NS1/Y3 | 0.1–10 | Lymph node | anti-lung carcinoma |

**Table II** Human hybridoma systems

| *Author/Fusion cell type* | *Derivation* | *Selection* | *Ig* | *Antibody* | $\mu g\ ml^{-1}$ |
|---|---|---|---|---|---|
| Croce *et al.*, (1980) GM-1500-6TGA1 | GM1500 | 6TG | $\kappa$ $\gamma$ | anti measles | — |
| Edwards *et al.*, (1982) LICR-LON-HMy2 | ARH77 | 6TG | $\kappa$ $\gamma$ | — | 0.1–1 |
| Kaplan & Olsson U266 AR1 (1980) | U266 | 8AG | $\varepsilon$ $\gamma$ | anti DNCB | 3–10 |
| Clark *et al.*, (1981) RPM1 8226 | RPM1 8226 | 0 | — | — | — |

6TG = 6 thioguanine; 8AG = 8 azaguanine.

were purified from peripheral blood using standard Ficoll–Paque techniques (Hutchins & Steel, 1979). Lymph nodes were teased apart by forceps and dead cells removed by Ficoll–Paque sedimentation. Intra-tumoural lymphocytes were collected from patients with poorly differentiated gliomas in a similar manner. Overall yields varied from 1–30 × $10^7$ viable lymphocytes.

*Cell fusion*

Cell fusion was carried out using polyethylene glycol (PEG) following the general procedure outlined by Hales (1977). An 8-azaguanine (8 AG) resistant human lymphoid cell line, LICR-LON-HMy2 (Edwards *et al.*, 1982), was used which was sensitive to hypoxanthine, aminopterin and thymidine (HAT) medium. This line was adapted for growth on serum-free medium and cloned. Several clones were tested for fusion with peripheral blood lymphocytes and one LICR-LON-HMy2/CAM1 (subsequently referred to as HMy2) chosen for further study. For each fusion, the recovered lymphocytes and a constant number of 5 × $10^7$ myeloma cells were suspended in serum-free Dulbecco's modified eagles medium (DMEM), mixed, and centrifuged at 1500 rpm for 10 min in a 50 ml conical-bottomed plastic centrifuge tube. The supernatant was drained off completely. Five hundred microlitres of PEG M.W. 1000, 41.7% (w/v), with 15% dimethyl sulphoxide (DMSO) in serum-free DMEM was added to the pellet, and the cells gently resuspended using the tip of the pipette. After 1 min, 0.5 ml of PEG 1000, 33% (w/v) in serum-free DMEM, but without DMSO, was added, and the mixture stirred gently for 3 min. Four millilitres of DMEM with 10% foetal calf serum (FCS) was added dropwise, and the mixture rocked for a further 4 min. Forty-five ml of DMEM with 10% FCS was slowly added, and the mixture was taken up carefully in a wide bore 25 ml pipette, and dispensed equally into each of 96 (2 ml) wells of 4 Linbro plates. One ml of DMEM with 10% FCS was added to each well, and renewed after 2 h. After 24 h, the medium was partly replaced by selected medium containing HAT 20% FCS (Miller & Ruddle, 1976). The selective medium was renewed daily for at least the first 2 weeks. Hybrid clones visibly appeared in some wells between 3–6 weeks after fusion. Supernatants from well-grown wells were taken for testing, supplemented with 10 mM Hepes buffer and 0.1% sodium azide, and stored at 4°C. Bulk supernatants from cloned hybrids growing in roller bottles were in Iscove's (Flow Laboratories) (Iscove & Melchers, 1978) serum-free medium supplemented with 5 $\mu$g ml$^{-1}$ of insulin. Bulk supernatants were harvested and concentrated using Millipore CX-10 (10,000 daltons exclusion) ultrafiltration unit.

*Immunoglobulin (Ig) assay*

Rabbit anti-human Ig antisera (Miles Laboratories) was diluted in phosphate buffered saline (PBS) to 1/1000. Fifty $\lambda$ aliquots were added to round-bottomed 96-well vinyl plastic plates and incubated overnight at 4°C. These plates were subsequently washed in medium containing Earle's buffered salt solution, 1% bovine serum albumin, 0.01% sodium azide, adjusted to pH 7.4 by 1 M sodium hydroxide. After washing 4 × by decanting the contents of the wells and replacing with a 50 $\mu$l of medium, the plates were left for 1 h at room temperature and again washed as previously described. Fifty $\mu$l of chain-specific monoclonal mouse anti-human Ig was added (Bethesda Research Laboratories) at a concentration of 1/5000. After 1 h incubation with the relevant monoclonal antibodies, the plates were washed and rat anti-mouse Ig coupled to $I^{125}$ using the chloramine-T method was added. After a final incubation of 1 h and 4 subsequent washes in complete medium, the plates were air dried, the wells cut with a hot wire, and counted in a $\gamma$ counter.

*Surface typing of hybridomas*

This was carried out by indirect immunofluorescence (Dorreen *et al.*, 1982). Cells were washed in acetate buffer (pH 5.5) to remove non-specifically adsorbed Ig and incubated in medium for 2 h. One million washed cells were incubated with monoclonal antibody against different lymphocyte subset antigens. These antibodies included the anti-Ig chain-specific McAbs (Bethesda Research Laboratories); anti-human $\beta$-2 microglobulin (clone 26/114 HLK, Sera Lab); anti-HLA (W6-32); anti-Ia (New England Nuclear, NE1/011); anti-Lyt3 reacting to T-lymphocytes (Becton Dickinson); OKT4 anti-inducer/helper T-lymphocytes (Ortho-Pharmaceuticals) OKT6, anticortical thymocytes (Ortho-Pharmaceuticals) and OKT8, anti-suppressor/cytotoxic T-lymphocytes (Ortho-Pharmaceuticals). After 1 h at 4°C the cells were washed in medium and then incubated with fluorescein isothiocyanate (FITC)-conjugated rabbit anti-mouse Ig (1:64, Miles). After a further 1 h at 4°C the cells were washed, suspended in 1% glycerol and examined. Surface Ig expression was quantified by flow cytometry. Cells were incubated for 1 h at 4°C with FITC-conjugated rabbit anti-human Ig, washed in EBSS containing 1% BSA, and examined on a flow cytometer.

*DNA content*

Hybridomas were cultured in 25 ml-tissue culture flasks in log phase. Cells were washed twice in EBSS containing 1% BSA and adjusted to $10^6$ cells ml$^{-1}$. Cells were pelleted and resuspended in ethidium bromide (0.05 mg ml$^{-1}$) in hypotonic (0.1%) sodium citrate. DNA histograms were obtained by means of a custom-built flow cytometer which incorporated an argon laser (Watson, 1981).

*Electron microscopy*

Five million cells were fixed in 3% glutaraldehyde in 0.1 M HEPES for 20 min. After washing in 0.1 M HEPES, cells were suspended in 1% osmium tetroxide in 0.1 M HEPES for 20 min, rewashed and suspended in 1% uranyl acetate for 30 min. After dehydration the cells were mounted in araldite blocks and examined by electron microscopy.

*Anti-tumour antibody activity*

A variety of human tumour cell lines were used to assess binding activity in an indirect radioimmunoassay. These included G/CCM glioma (gift of I. Freshney) HT29 colorectal carcinoma (from J. Fogh); MOR lung adenocarcinoma (M. Ellison); Calu-1 squamous cell lung carcinoma (J. Fogh); MCF-7 (P. Rudland); and the MRC5 fibroblast line. Freshly trypsinised cells were washed 3 × by centrifugation and suspended in assay medium identical with that used for Ig typing. Viable cells were counted by trypan-blue exclusion and adjusted to a concentration of $2 \times 10^6 ml^{-1}$. Fifty $\mu l$ of cell suspension was placed into each well of round-bottomed Cooke microtitre plates which had been previously incubated overnight with $50 \mu l$ well of poly L-lysine in phosphate buffered saline at 37°C. The cells were fixed for 1 h in 0.25% glutaraldehyde and washed 3 × in medium. Fifty $\mu l$ of supernatant was added and incubated at room temperature for 1 h. After washing three times $50 \mu l$ of mouse anti-human light chain antibody at a dilution of 1/5000 was added and incubated for 1 h. The final stage of the assay was the addition of $50 \mu l$ radioiodinated rabbit anti-mouse Ig. After a further hour the cells were washed and counted on a $\gamma$ counter.

### Results

### *Cell fusion*

Clinical material was obtained from 180 patients with a variety of tumours over the course of 1 year (Table III). Apparently successful initial fusion was observed in 55 patients. Low lymphocyte yields and infection were the major problems in the early stages. Infection was a particular hazard in samples from patients with colorectal carcinoma where lymph nodes and contaminated large bowel were placed in a single sterile container for delivery to the laboratory. Cloned hybrids were obtained in 24 patients. Hybrids appeared between 4–8 weeks following fusion and were seen as clumps of piled-up cells amongst the debris of dying normal lymphocytes and HMy2 cells (Figure 1). Once established, hybridomas were rapidly growing with doubling times of 24–36 h. All hybrids tested were easily adapted for growth in serum-free (Iscoves) medium in 41 roller bottles.

### *Ig secretion*

All hybridomas continued to produce the $\kappa$ and $\gamma$ chains secreted by HMy2. In addition, 14% produced $\lambda$ chains, 15% $\mu$ chains, 13% $\alpha$ chains and 1% $\varepsilon$ chains (Table 4). The production of new Igs continued after prolonged tissue culture. One–$5 \mu g \, Ig \, ml^{-1}$ was detected in the supernatants.

### *Lymphocyte antigens and human hybridomas*

Table V outlines the results of indirect immunofluorescence using a set of commercially available McAbs to lymphocyte differentiation antigens. Peripheral blood lymphocytes from a normal donor showed partial reactivity to all typing reagents. HMy2 contained surface $\kappa$ and $\gamma$ Ig, as

**Table III** Human hybridoma production

| *Tumour* | *Samples produced* | *Lymphocyte origin* | *Successful fusion* | *Patients with hybrids* | *Total hybrids* |
|---|---|---|---|---|---|
| Lung | 14 | RN | 8 | 2 | 27 |
| Breast | 29 | RN | 13 | 4 | 28 |
| Colorectal | 42 | RN | 12 | 3 | 6 |
| Glioma | 39 | IT | 18 | 9 | 84 |
| Kidney | 3 | RN | 0 | 0 | 0 |
| Sarcoma | 2 | IT | 0 | 0 | 0 |
| Melanoma | 2 | RN | 1 | 1 | 1 |
| Uterus | 13 | RN | 5 | 2 | 7 |
| Stomach | 14 | RN | 5 | 1 | 7 |
| Bladder | 18 | RN | 0 | 0 | 0 |
| Burkitts | 4 | PB | 3 | 2 | 2 |

RN regional node.
IT intratumoural
PB peripheral blood

**Table IV** Human immunoglobulins produced by hybridomas

| Tumour | Total hybrids | Ig secreted $\kappa$ | $\lambda$ | $\gamma$ | $\mu$ | $\alpha$ | $\varepsilon$ |
|---|---|---|---|---|---|---|---|
| Lung | 27 | 27 | 4 | 27 | 6 | 1 | 1 |
| Breast | 28 | 28 | 3 | 28 | 5 | 4 | 0 |
| Colorectal | 6 | 6 | 1 | 6 | 1 | 0 | 0 |
| Glioma | 84 | 84 | 13 | 84 | 11 | 15 | 2 |
| Melanoma | 1 | 1 | 0 | 1 | 0 | 0 | 0 |
| Uterus | 7 | 7 | 2 | 7 | 1 | 0 | 0 |
| Stomach | 7 | 7 | 0 | 7 | 0 | 1 | 0 |
| Burkitts | 2 | 2 | 0 | 2 | 0 | 0 | 0 |

**Table V** Lymphocyte antigens on human hybridomas

| | PBL | HMy2 | Hybridomas |
|---|---|---|---|
| $\alpha$Ig$\kappa$ | + | + | + |
| $\alpha$Ig$\lambda$ | + | − | ± |
| $\alpha$Ig$\gamma$ | + | + | + |
| $\alpha$Ig$\mu$ | + | − | ± |
| $\alpha$Ig$\alpha$ | + | − | ± |
| $\alpha\beta_2$m | + | + | + |
| $\alpha$HLA | + | + | + |
| $\alpha$Ia | + | + | + |
| $\alpha$Lyt 3 | + | − | − |
| OKT4 | + | − | − |
| OKT6 | + | − | − |
| OKT8 | + | − | − |

**Figure 1** Human hybridoma cells growing in HAT. Debris of dying lymphocytes and HMy2 cells in background.

well as β-2 microglobulin, HLA and IA. No T-cell markers were noted. A set of 20 hybridomas were typed. Surface Ig expression corresponded with detected chains secreted into the supernatant. All hybridomas expressed β-2 microglobulin, HLA and IA. No T-cell antigens were expressed in any of the hybridomas tested. Figure 2 shows the flow cytometry analysis of surface Ig content detected by polyclonal rabbit anti-human Ig. Peripheral blood lymphocytes show a slight increase over the HMy2 content of surface Ig. All hybrids tested show a considerable increase in the amount of Ig expressed.

**Figure 2** Surface Ig expressed on lymphocytes, HMy2 cells and 3 hybridomas detected using FITC-anti human Ig and flow cytometry.

### *DNA content*

Figure 3 shows the DNA content of lymphocytes, HMy2 and one hybridoma. The DNA content of the hybrid cell is approximately the sum of that of the parent myeloma, plus that of the lymphocytes. After 8 months of continuous culture, no change was observed in this increased DNA content.

### *Electron microscopy*

Figure 4 shows electron microscopy of HMy2 and a resultant hybridoma, LGL1-1D6. The endoplasmic reticulum was poorly developed.

### *Anti-tumour antibody activity and specificity*

Antibodies were initially screened for binding activity against the cell line most appropriate to the source of donor lymphocyte. An initial screen of 40 hybridoma supernatants is shown in Figure 5. Despite the sensitivity of the binding assay, the counts bound were low. After concentration, however, (Figure 6), titration curves were obtained. These curves show only weak binding of antibody. However the binding was significantly above background when compared to that of HMy2 Ig at similar concentration (Figure 6). The specificity of binding was determined using several tumour cell lines (Table VI). Peripheral blood lymphocytes and red blood cells from normal donors were used as controls. It can be seen that all the antibodies isolated so far bind to a variety of cell types and are not individually tumour-specific. More precise definition of specificity was attempted using immunohistological methods on fresh frozen biopsies of tumour and normal tissue. No significant binding above background was observed despite clear immunofluorescence patterns with cell lines.

### Discussion

In this paper we have demonstrated that lymphocytes from patients with several tumour types can be successfully fused with a human myeloma line to produce stable hybridomas. These hybridomas secrete Igs, several of which have been found to show weak binding to tumour cell lines in an indirect radioimmunoassay. Evidence for hybridisation, rather than outgrowth of lymphoblastoid lines from the patients lymphocytes, is provided by the continued secretion of HMy2 Ig as well as the new lymphocyte Ig in cloned hybridomas. Flow cytometric DNA analysis shows the stably increased DNA content characteristic of hybrids. Formal karyotypic analysis has been performed on hybridomas derived from HMy2

**Figure 3** DNA content of lymphocytes, HMy cells and LGL1-1D6 hybridoma.

which confirm a stably increased chromosome number (Edwards *et al.*, 1982).

There are several problems with the LICE-LON-HMy2 hybridoma system. Firstly, the low fusion frequency results in a large workload to produce small numbers of hybrids. Several methods have been used in attempts to increase this frequency. These have included provision of feeder cells and secondary *in vitro* stimulation by either antigen (tumour cells) or by pokeweed mitogen. No increase in fusion frequency was observed. A second problem is the continued secretion of the HMy2 $\kappa$ and $\gamma$ immunoglobulin chains by the hybridomas. A true assessment of the numbers of hybrids secreting Ig coded for by genes of the donor lymphocytes is thus difficult. Furthermore, mixed antibody molecules comprised of chains coded by both myeloma or lymphocyte genes will occur. Such molecules will have reduced antibody activity. A third problem is the low antibody secretion rate of between 1 and $5\,\mu g\,ml^{-1}$. Large amounts of tissue culture supernatant must be concentrated in order to increase signal-to-noise ratio in the radioimmunoassay. The poorly developed endoplasmic reticulum in all hybrids examined by electron microscopy suggests that the rate of protein synthesis may well be limiting. The initial screen for anti-tumour antibody activity may be unable to detect the small amounts of weak antibodies. Finally, the low anti-tumour binding activity hinders the analysis of specificity of the resulting antibodies. A combination of low secretion

**Figure 4** Electron microscopy of HMy2 and LGL1-1D6 (×4000).

**Figure 5** Initial screening assay for anti-tumour binding activity. Forty supernatants tested on GCCM—a rapidly-growing human glioma line.

**Figure 6** Concentrated supernatants (×40) titrated in binding assay against GCCM. ○——○ LGL1-1D6; △——△ LGL10-3B5; ●——● HMy2 sup.

rate, mixed Ig chain molecules, and low affinity may all contribute to this low binding. Our attempts to produce a high secreting variant of HMy2 by dilution cloning and subsequent assay have so far been unsuccessful.

The specificity studies on 12 antibodies clearly show a wide range of weak binding to different tumour cell lines. It is of interest that none of the antibodies bound to the benign fibroblast line (MRC5), normal red cells, or peripheral blood lymphocytes. Our attempts to localise the antigens by immunohistological techniques have so far been unsuccessful due to a combination of weak binding and high background Ig in human tissues. We are currently using immunochemical methods to detect the presence of the recognised antigens on a variety of human tissue types. Despite the apparent lack of specificity to individual tumours, human monoclonal antibodies may well have significant diagnostic and therapeutic implications in clinical oncology.

We would like to thank the many physicians, surgeons and pathologists who have contributed biopsy specimens. We also thank Dr. P. Edwards and Dr. M. O'Hare for providing LICR-LON-HMy2 and for much helpful discussion; Mr. N. Thomson for electron microscopy; Dr. J. Habeshaw for initial lymphocyte marker studies and Dr. A. Levine for Burkitt lymphoma lymphocytes.

**Table VI** Binding of human hybridoma supernatants to various human cell lines, peripheral lymphocytes and red cells

| *Supernatant* | *G/CCM* | *HT29* | *MOR* | *CALU* 1 | *MCF* 7 | *MRC* 5 | *PBL* | *RBC* |
|---|---|---|---|---|---|---|---|---|
| Glioma | | | | | | | | |
| LGL1—1C3 | +++ | ++ | + | − | +++ | − | − | − |
| LGL1—1C6 | +++ | ++ | + | − | ++ | − | − | − |
| LGL1—1D6 | ++ | + | ++ | + | + | − | ± | − |
| LGL1—2C1 | ++ | ++ | + | + | − | − | − | ± |
| LGL7—1A2 | ++ | + | + | − | + | ± | − | ± |
| LGL7—3A2 | ++ | + | + | ++ | − | − | − | − |
| LGL10—3B5 | ++ | + | + | − | ++ | − | − | − |
| LGL22—4D6 | ++ | + | ++ | + | + | − | − | ± |
| Lung | | | | | | | | |
| LLU1—3D1 | ++ | ++ | ++ | ++ | + | − | − | ± |
| LLU6—1A1 | ++ | + | ++ | ++ | + | ± | − | ± |
| LLU6—2A4 | ++ | + | + | + | − | − | − | − |
| LLU6—3D4 | ++ | + | ++ | ++ | + | − | − | − |

− 0–50 c.p.m.

± 50–100

+ 100–150

++ 150–200

+++ >200

} above HMy2 supernatant background

### References

BROWN, J.P., WOODBURY, R.G., HART, C.E., HELLSTROM, I. & HELLSTROM, K.E. (1981). Quantitative analysis of melanoma associated antigen p. 97 in normal and neoplastic tissues. *Proc. Natl Acad. Sci.*, **78**, 539.

CLARK, S.A., STIMSON, W., WILLIAMSON, A.R. & DUCK, H.M. (1981). Hybridoma production by a human myeloma using biochemical selection. *J. Supramol. Struct. Cell. Biochem.*, (Suppl.) **5**, 100a.

CROCE, C.M., LINNENBACH, A., HALL, W., STEPLEWSKI, Z. & KOPROWSKI, H. (1980). Production of human hybridomas secreting antibodies to measles virus. *Nature*, **288**, 488.

DORREEN, M.S., HABESHAW, J.A., WRIGLEY, P.F.M. & LISTER, T.A. (1982). Distribution of T-lymphocyte subsets in Hodgkin's disease characterised by monoclonal antibodies. *Br. J. Cancer*, **45**, 491.

EDWARDS, P.A.W., SMITH, C.M., NEVILLE, A. & O'HARE, M.J. (1982). A human–human hybridoma system based on a fast-growing mutant of the ARH-77 plasma cell leukaemia-derived line. *Eur. J. Immunol.*, **12**, 641.

HALES, A. (1977). A procedure for the fusion of cells in suspension by means of polyethylene glycol. *Somatic Cell Genet.*, **3**, 227.

HUTCHINS, D. & STEEL, C. (1979). Separation of human lymphocytes on the basis of volume and density. In *Separation of Cells and Subcellular Elements*. (Ed. Peeters). Oxford: Pergamon Press, p. 28.

ISCOVE, N.N. & MELCHERS, F. (1978). Complete replacement of serum by albumin transferrin and soy bean lipid in cultures of LPS-reactive B lymphocytes. *J. Exp. Med.*, **147**, 923.

KAPLAN, H. & OLSSON, L. (1980). Human–human hybridomas producing monoclonal antibodies of predefined specificity. *Proc. Natl Acad. Sci.*, **77**, 5429.

KOPROWSKI, H., HERLYN, M., STEPLEWSKI, Z. & SEARS, H. (1981). Specific antigen in serum of patients with colon carcinoma. *Science*, **212**, 53.

LENNOX, E.S. & SIKORA, K. (1982). Definition of human tumour antigens. In *Monoclonal Antibodies in Clinical Medicine* (Eds. McMichael & Fabre) London: Academic Press, p. 111.

LEVY, R., DILLEY, J., SIKORA, K. & KUCHERLAPATI, R. (1978). Hybrids of normal and malignant T and B lymphocytes. *Curr. Top. Microbiol. Immunol.*, **81**, 170.

MACH, J.P., BUCHEGGER, F., GIRARDET, C. & 7 others (1982). Uses of radiolabelled monoclonal anti-CEA antibodies for the detection of human carcinomas by external photoscanning and tomoscintigraphy. *Immunol. Today*, **2**, 239.

MARX, J.L. (1982). Monoclonal antibodies in cancer. *Science*, **216**, 283.

MILLER, R.A., MALONEY, D.G., WARNKE, R. & LEVY, R. (1982). Treatment of B-cell lymphoma with monoclonal anti-idiotype antibody. *N. Engl. J. Med.*, **306**, 517.

MILLER, R.A. & RUDDLE, F.G. (1976). Pluripotent teratocarcinoma—thymus somatic cell hybrids. *Cell*, **9**, 45.

NOWINSKI, R., BERGLUND, C., LANE, J., LOSTROM, M., BERNSTEIN, I., YOUNG, W., HAKOMORI, S., HILL, L. & COONEY, M. (1980). Human monoclonal antibody against Forsmann antigen. *Science*, **210**, 537.

SCHLOM, J., WUNDERLICH, D. & TERAMOTO, U.A. (1980). Generation of human monoclonal antibodies reactive with human mammary carcinoma cells. *Proc. Natl Acad. Sci.*, **77**, 6841.

SCHWABER, J. & COHEN, E.P. (1974). Pattern of Ig synthesis and assembly in a human–mouse somatic cell hybrid clone. *Proc. Natl Acad. Sci.*, **71**, 2203.

SEARS, H.F., ATKINSON, B., MATTIS, J. & 5 others. (1982). Phase I clinical trial of monoclonal antibody in treatment of gastrointestinal tumour. *Lancet*, **i**, 762.

SHIKU, H., TAKAHASTI, T., REDNICK, L., OETTGEN, H. & OLD, L.J. (1977). Cell surface antigens on human malignant melanoma. *J. Exp. Med.*, **145**, 784.

SIKORA, K. & WRIGHT, R. (1981). Human monoclonal antibodies to lung cancer antigens. *Br. J. Cancer*, **43**, 696.

WATSON, J.V. (1981). Dual laser beam focussing for flow cytometry through a single crossed cylindrical lens pair. *Cytometry*, **1**, 14.

WUNDERLICH, D., TERATOMO, Y.A., ALFORD, C. & SCHLOM, J. (1981). Use of lymphocytes from axillary lymph nodes of mastectomy patients to generate human monoclonal antibodies. *Eur. J. Cancer*, **17**, 719.